# Induction of liver hypertrophy for extended liver surgery and partial liver transplantation: State of the art of parenchyma augmentation–assisted liver surgery

**DOI:** 10.1007/s00423-021-02148-2

**Published:** 2021-03-19

**Authors:** Philip C. Müller, Michael Linecker, Elvan O. Kirimker, Christian E. Oberkofler, Pierre-Alain Clavien, Deniz Balci, Henrik Petrowsky

**Affiliations:** 1 Swiss HPB and Transplantation Center, Department of Surgery and Transplantation, University Hospital Zurich, Zurich, Switzerland; 2 Department of Surgery and Transplantation, University Medical Center Schleswig-Holstein, Campus Kiel, Kiel, Germany; 3 Department of Surgery and Liver Transplantation Unit, Ankara University School of Medicine, Ankara, Turkey

**Keywords:** Liver surgery, Liver augmentation, Portal vein embolization, Transarterial chemoembolization, Two-staged hepatectomy, Associating liver partition and portal vein ligation for staged hepatectomy

## Abstract

**Background:**

Liver surgery and transplantation currently represent the only curative treatment options for primary and secondary hepatic malignancies. Despite the ability of the liver to regenerate after tissue loss, 25–30% future liver remnant is considered the minimum requirement to prevent serious risk for post-hepatectomy liver failure.

**Purpose:**

The aim of this review is to depict the various interventions for liver parenchyma augmentation–assisting surgery enabling extended liver resections. The article summarizes one- and two-stage procedures with a focus on hypertrophy- and corresponding resection rates.

**Conclusions:**

To induce liver parenchymal augmentation prior to hepatectomy, most techniques rely on portal vein occlusion, but more recently inclusion of parenchymal splitting, hepatic vein occlusion, and partial liver transplantation has extended the technical armamentarium. Safely accomplishing major and ultimately total hepatectomy by these techniques requires integration into a meaningful oncological concept. The advent of highly effective chemotherapeutic regimen in the neo-adjuvant, interstage, and adjuvant setting has underlined an aggressive surgical approach in the given setting to convert formerly “palliative” disease into a curative and sometimes in a “chronic” disease.

## Introduction

Liver surgery and transplantation currently represent the only curative treatment options for primary and secondary hepatic malignancies [1]. Despite the unique ability of the liver to regenerate after major tissue loss, 25–30% future liver remnant (FLR) is considered the minimum requirement for patients without underlying liver disease [2]. However, major hepatectomies are associated with a serious risk for post-hepatectomy liver failure (PHLF) especially in the scenario of underlying liver disease. Patients with extensive colorectal liver metastases (CRLM) or large hepatocellular carcinoma (HCC) often present with locally unresectable hepatic disease at the time of diagnosis due to a too small FLR, which is often the barrier for a curative approach.

A variety of developments have been introduced over the last decades to augment the volume of the FLR facilitating most extended liver resections. Exactly 100 years ago, Rous and Larimore first reported on the effect of portal vein occlusion in a rabbit model [3]. After left portal vein ligation (PVL), they observed hypertrophy of the contralateral and atrophy of the ipsilateral liver within 3 months. At that time, the intentional use of PVL to induce liver growth has not been considered, as major blood loss was still the main obstacle limiting hepatic surgery, which was still responsible for the majority of procedure-related mortalities. It took until 1975, when Honjo et al. reported the first clinical case of PVL for unresectable liver cancer.[4] In 1984, Makuuchi et al. introduced the principle of portal vein occlusion–induced liver augmentation into clinical practice [5]. Instead of PVL, he applied preoperative transcatheter embolization of portal branches in analogy to tumor-induced occlusion of the portal vein. In the inaugural series, portal vein embolization (PVE) was performed in 14 patients with hilar cholangiocarcinoma, in whom an extended hepatectomy was successfully performed 6 to 41 days after PVE [6]. Further steps included the introduction of intentionally planned two-staged hepatectomies (TSH) utilizing PVL and PVE for liver parenchyma augmentation to treat previously unresectable multiple metastases. The main drawback of PVE/PVL is a relatively long waiting period between liver growth induction and liver resection including the pending hazard of tumor progression. In addition, a failure to grow is not infrequently observed rendering resection rates well below 50%. To overcome these limitations, new treatment concepts such as associating liver partition with portal vein ligation for staged hepatectomy (ALPPS), combined PVE/hepatic vein embolization (HVE), and resection and partial liver segment 2/3 Transplantation with delayed total hepatectomy (RAPID) were recently introduced. The aim of this review is to present the different strategies of parenchyma augmentation–assisted liver surgery with its main indications, rationale, and limitations.

## Definition of parenchyma augmentation–assisted liver surgery

We define parenchyma augmentation–assisted liver surgery as any hepatobiliary surgery or liver transplantation procedure, which integrates means of liver parenchyma augmentation for a too small FLR or partial graft in order to prevent PHLF or small-for-size syndrome, and thus perform safe surgery. Interventions for tissue augmentation can be either applied before surgery in one-stage procedures such as preoperative PVE or are an intraoperative element at stage-one surgery of two-stage procedures including conventional TSH, ALPPS, or RAPID procedures.

## One-stage hepatectomy with portal vein embolization (+ segment IV embolization)

**Fig. 1:**
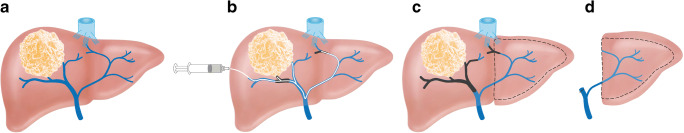
Patient with a large single tumor potentially resectable by single stage hepatectomy but with a small FLR (**A**). Portal vein embolization of the tumor-bearing side can be used for tissue augmentation (**B**). After sufficient hypertrophy (**C**), typically 4–8 weeks after embolization, completion hepatectomy is performed (**D**)

Makuuchi et al. introduced PVE in the setting of perihilar cholangiocarcinoma (PHC) based on the principle that occlusion of a portal branch leads to an ipsilateral atrophy and a subsequent hypertrophy of the contralateral lobe (Fig. 1) [5, 6]. The concept was developed to increase the resection rate and minimizing the risk of PHLF after extended resections in PHC [7–9].

A large meta-analysis from 2008 including 1088 patients showed an FLR augmentation after transileocolic PVE of 9.7% versus 12% after percutaneous transhepatic PVE. Post-PVE morbidity was low (2.2%) without any post-interventional mortality. Completion hepatectomy could be achieved in 85% of the patients 29 days after PVE. Main reasons for non-resection following PVE were progression of liver metastasis (*n* = 43), extrahepatic spread (*n* = 35), and inadequate hypertrophy of remnant liver (*n* = 18). In the postoperative course, 2.5% of patients had transient liver failure and 0.8% experienced acute liver failure leading to death [10]. In 2018, Wajswol et al. performed a meta-analysis of 18 articles with 607 patients undergoing PVE for major hepatic resections [11]. The technical success rate was 99% and the percutaneous transhepatic PVE was the most common approach (contralateral *n* = 417, ipsilateral 132). Major complications after PVE occurred in 19 patients (3.1%). The time interval between PVE and volume assessment was highly variable and ranged from 2 to 10 weeks. The authors found a relative hypertrophy rate of 49%. Liver resection was finally performed in 461 of 607 patients (76%). Similarly to the previous meta-analysis, main reasons for non-resection were disease progression (*n* = 114) and insufficient FLR hypertrophy (*n* = 24). Postoperative liver insufficiency occurred in 21 patients (4.5%) [11].

Aiming for a right trisectionectomy, segment IV portal branches from the left portal system may be embolized in addition to the embolization of the right portal trunk. This maneuver potentially provides a greater volume augmentation of the FLR [12–15] but carries the inherent risk of injuring the left portal vein. Furthermore, embolization material may inadvertently find its way into the left portal venous system, leading to portal venous thrombosis of the FLR [16]. For this reason, selective embolization of segment IV is only advisable when a dedicated interventional expert radiologist is available. However, based on limited evidence so far, no difference in PVE associated complications between right PVE and right PVE + segment IV could be detected. In contrast, volume augmentation in patients undergoing right PVE + segment IV was markedly higher with an increase in FLR size ranging from 47 to 54% as compared to 26 to 38% for right PVE without segment IV [12–14].

## One-stage hepatectomy with portal vein- and hepatic vein embolization

**Fig. 2:**
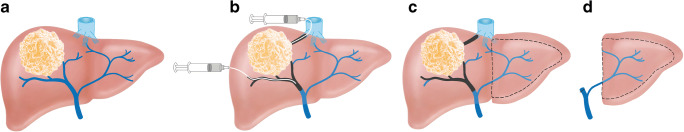
Patient with a large single tumor (**A**) that is amenable to a single stage hepatectomy, but has an insufficient FLR requires tissue augmentation by combined portal vein- and hepatic vein embolization of the tumor-bearing side (**B**). After sufficient hypertrophy (**C**), typically 4–8 weeks after embolization, completion hepatectomy is performed (**D**)

Compared to PVE alone, combined PVE and hepatic vein embolization (HVE) is assumed to accelerate liver growth of the contralateral liver (Fig. 2). The technique is also referred to as liver venous deprivation [17], and more recently, as radiological simultaneous portohepatic vein embolization (RASPE) [18]. For simplicity, PVE/HVE will be used in this review. From experience in living donor liver transplantation, it is well known that there is a strong inverse relationship between hepatic congestion and liver regeneration [19]. While regeneration of congested liver parts is impaired ultimately leading to atrophy, parts with preserved outflow will experience increased regeneration. Although the exact pathophysiologic mechanism behind regeneration after PVE/HVE is not fully understood, HVE theoretically decreases the hepatic buffer response of increased hepatic arterial inflow following PVE [17, 20, 21]. Furthermore, the development of portovenous collaterals to the deportalized liver seems to occur early after PVE, but not in PVE/HVE. In a porcine model, portal vein perfusion stayed limited to the non-embolized segments after PVE/HVE, while 7 days after PVL alone the entire liver was reperfused with portal blood. This suppression of portovenous collaterals in PVE/HVE is a potential explanation of accelerated volume augmentation [21].

Sequential HVE after insufficient liver regeneration after PVE was first described in 2009 by Hwang et al. in 12 patients. In 9 patients undergoing hepatectomy, the FLR before PVE was 35%, 2 weeks after PVE 40%, and 2 weeks after HVE 44% [22]. Compared to PVE alone, this sequential approach did not reduce the time to resection and therefore as well not the drop-out of patients from potentially curative resection. In order to eliminate the waiting time between PVE and HVE, Guiu et al. introduced the concept of simultaneous PVE and HVE in 2016. In their initial report, seven patients successfully underwent PVE/HVE. After a mean time of 23 days (13–30 days), the FLR increased from 28 to 41% and resection was achieved in 6/7 patients [17].

To achieve combined PVE/HVE, the right hepatic vein (HV) is accessed under ultrasound guidance, then the right PV is accessed using the same technique. PVE is performed with iodized oil (e.g., lipiodol) and n-butyl-cyanoacrylate, without segment IV embolization. Then an Amplatzer Vascular II Plug is deployed in the right HV. In the original description, the authors furthermore checked for distal branches of the right HV and potential veno-venous collaterals. Embolization of these branches was then performed using a lipiodol-n-butyl-cyanoacrylate mixture [17, 23].

Since the first publication, seven further reports including between 6 and 37 patients have been published [17, 18, 23–28]. PVE/HVE was mainly used to treat CRLM (55%); however, most studies included both primary and secondary liver tumors [29]. No serious adverse events and especially no extensive liver necrosis has been reported after PVE/HVE thus far. Therefore, arterial perfusion seems to be sufficient in the embolized hemi-liver. The interval between embolization and hepatic resection ranged from 21 to 45 days. Compared to PVE alone, an accelerated liver growth is postulated after PVE/HVE. However, a comparison of clinical data is sometimes difficult due to the use of different metrics to quantify hypertrophy. Compared to the baseline FLR volume, a hypertrophy of 35–60% was observed after PVE/HVE [18, 23, 27, 28]. The hypertrophy of the liver resulted in resection rates between 67 and 100% [26, 28], while the latest review reported an overall resection rate of 87% [29]. Disease progression was the reason for drop out in 16/17 patients. Despite the limited evidence at the moment, there are currently two prospective trials recruiting. The HYPER-LIV01 trial (NCT03841305) is a randomized single-center trial from France [30], while the DRAGON trial (NCT04272931) is an international prospective multicenter trial.

## One-stage hepatectomy with sequential transarterial chemoembolization and portal vein embolization in HCC

**Fig. 3:**
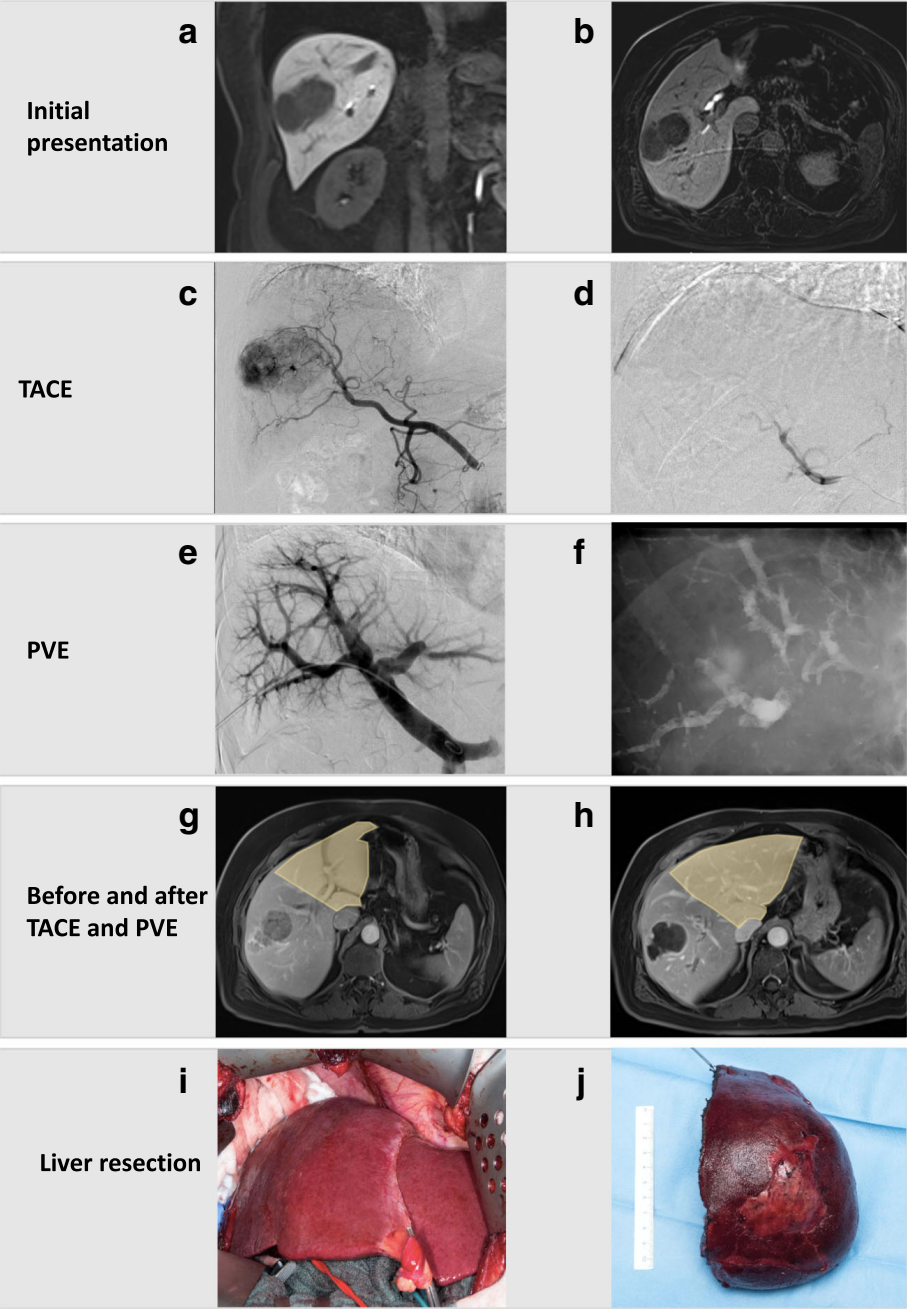
Clinical case of transarterial chemoembolization and sequential portal vein embolization (University of Zurich). This 69-year-old patient presented with a 7-cm hepatocellular carcinoma (HCC) in liver segments V/VIII and an alpha-fetoprotein (AFP) of 42.649 μg/l (**A**/**B**). No portal hypertension or liver cirrhosis was present. For a right hemihepatectomy, a sFLR of 27% was calculated. Since the indocyanine green (ICG) test showed impaired liver function with a plasma disappearance rate (PDR) of 12.1%/min (normal value >18%/min) and ICG retention rate (R_15_) of 14.7% (normal value <10%), we opted for transarterial chemoembolization (**C**/**D**)followed by portal vein embolization 2 weeks later (**E**/**F**). After the successful embolization, the AFP dropped to 209 μg/l and the sFLR increased to 39% (**G**/**H**). We then performed a right hemihepatectomy (**I**/**J**). Three months postoperatively, the patient is tumor-free and in a good general condition

**Fig. 4:**
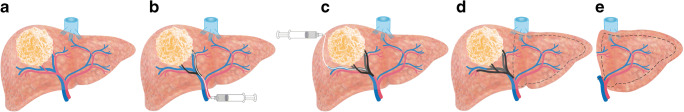
In patients with large hepatic tumors, underlying liver disease, and small FLR (**A**), portal vein embolization can be combined with sequential transarterial chemoembolization. For this approach, selective catheterization of the right hepatic artery is performed, then chemotherapy is infused into the selected feeding artery (**B**). Two to 4 weeks later, this procedure is followed by portal vein embolization (**C**). After sufficient hypertrophy (**D**), completion hepatectomy is performed (**E**)

Major hepatic resection in HCC patients is often more challenging than for other hepatic malignancies due to the underlying liver disease or cirrhosis, which are associated with a higher risk of hepatic failure.[31] Compared to PVE in healthy livers, the degree of PVE-triggered hypertrophy in chronic liver disease is less predictable and PVE might theoretically induce a compensatory increase in hepatic arterial flow in the embolized segments [32]. Therefore, sequential transarterial chemoembolization (TACE) and PVE was proposed to control HCC, thereby buying time to augment liver volume (Fig. 3). For TACE, selective catheterization of the right hepatic artery is performed, then chemotherapy and an emulsion of iodized oil (e.g., lipiodol) is infused into the selected feeding artery. Afterwards, embolization with microspheres (e.g., absorbable gelatin sponge particles) is performed until arterial stasis is achieved (Fig. 4). TACE has antitumor effects by occluding tumor feeding vessels and showed promising results in HCC patients with chronic liver disease with a tumor response in half of the patients [33–36]. Evaluating the volumetric changes, different studies showed that sequential TACE and PVE induced even a greater hypertrophy in the non-embolized liver than PVE alone (Table 1) [34, 38, 39]. The time interval from TACE to PVE was 30 days (range 9–120 days) and from PVE to surgery 28 days (range 21–45 days) in a systematic review [40]. In retrospective studies, TACE + PVE led to a greater hypertrophy [34, 35, 38, 39] with improved overall survival (OS) [35, 39] and disease-free survival (DFS) [34, 35, 38, 39] as compared to PVE alone. However, the advantages of TACE need to be carefully balanced against its adverse events. Detoriating liver function, ischemic cholangitis, and intrahepatic abscess have been repeatedly reported [33, 37, 41]. On the other hand, failure to achieve sufficient hypertrophy after TACE and PVE indicates an impaired capacity of the liver to regenerate in patients with chronic liver disease. This feature can be used as “dynamic” liver function test to identify patients who are eligible for surgery.

**Table 1:** Studies comparing patients undergoing TACE + PVE with PVE alone

Reference	Study design	Year	Strategy	Patients (*n*)	Volume increase* (%)	Time° (d)	Resection rate (%)
Aoki et al.[37]	RCS	2004	TACE + PVE	17	22	9 + 25	94
Ogata et al.[38]	RCS	2006	TACE + PVE	18	12	25 + 37	-
			PVE	18	8	40	-
Yoo et al.[35]	RCS	2011	TACE + PVE	71	7	36 + 29	96
			PVE	64	6	31	91
Peng et al.[36]**	RCS	2012	TACE + PVE	29	7	-	93
			PVE	25	8	-	76
Terasawa et al.[34]	RCS	2019	TACE + PVE	23	43	47 + 47	92
			PVE	28	31	48	68
Park et al.[39]	RCS	2020	TACE + PVE	109	18	75	-
			PVE	38	12	23	-

*PVE*, portal vein embolization; *RCS*, retrospective case series; *TACE*, transarterial chemoembolization

*Future liver remnant

**Including secondary liver tumors

° Time interval between interventions and the surgical resection

## Conventional two-stage hepatectomy with portal vein ligation or portal vein embolization

**Fig. 5:**
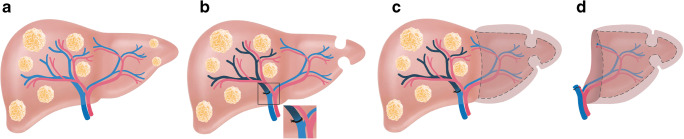
In case of extensive bilobar tumorload (**A**), a two-staged approach may be necessary. This can be a conventional TSH with portal vein ligation and clearing of the FLR in a first step (**B**). After an interstage interval of 4–8 weeks (**C**), completion hepatectomy is performed after sufficient hypertrophy of the FLR (**D**)

In 2000, Adam et al. introduced the concept of TSH to treat previously unresectable multiple CRLM due to insufficient FLR. In a first stage, the highest possible number of metastases were removed, while the intention of this first operation was not clearance of all metastases. After sufficient hypertrophy with an interval of 2–14 months, 13 of 16 patients (81%) underwent successful hepatectomy to remove the remaining tumors [42]. Since the aforementioned concept still had a considerable risk of PHLF, Jaeck et al. combined TSH with PVE. In a first step, clearing of the hemi-liver with less tumor load (most often the left side) was performed by resection and/or ablation, followed by PVE immediately after stage 1 surgery and second-stage hepatectomy after sufficient augmentation of the FLR, usually after 5–8 weeks (Fig. 5). This concept was successfully applied in 25 of 33 patients (76%) with CRLM without perioperative mortality and a 1- and 3-year survival rate of 70% and 54% [43].

To minimize the number of procedures and the associated risks, the Belghiti group suggested simultaneous portal vein ligation (PVL) during the clearing of the left hemi-liver as a surgical variant of portal vein occlusion [44–46]. A recent meta-analysis including 21 studies compared 1953 PVE and 123 PVL patients. The authors found no difference in terms of FLR hypertrophy (PVE 43% vs. PVL 39%), although there was a high variability in time interval between PVE/PVL and hypertrophy evaluation. Post-interventional morbidity after PVE (3.9%) and PVL (5.2%) was comparable. However, the number of uncompleted two-stage resections due to inadequate hypertrophy was lower after PVL (0%) vs. PVE (4.3%) [47].

Outcomes of TSH for bilateral CRLM are shown in Table 2. Completion rates for both stages range between 60 and 100% [48, 50, 51, 53], with FLR hypertrophy between 30 and 60%[43, 48, 50, 57] while postoperative mortality and morbidity rates were between 0 and 15% [42, 43, 48] and 20–59% [48–50, 56]. A 3- and 5-year OS of 33–84% and 32–64% was reported [48, 52, 56, 59, 60]. These data demonstrate comparable morbidity with one-stage hepatectomies and favorable oncologic outcomes for well-selected patients with multiple, bilobar CRLM. More recently, either the first or both steps of TSH have been performed laparoscopically to further diminish surgical trauma [58, 61, 62]. The comparative study by Okumura et al. showed a reduction of postoperative complications (32 vs. 60%; *P* = 0.047), a shorter hospital stay (9 vs. 16 days; *P* = 0.013) and earlier administration of chemotherapy (1.6 vs. 2 months; *P* = 0.039) in favor for the laparoscopic approach [62].

**Table 2:** Perioperative patient outcomes of patients undergoing two-staged hepatectomies

Reference	Year	Patients (*n*)	PVE/PVL (%)	Volume increase* (%)	Time° (d)	Resection rate (%)	Morbidity (%)	Mortality (%)
Adam et al. [42]	2000	16	44	-	120	81	38	15
Jaeck et al. [43]	2004	33	100	31	-	76	56	0
Tanaka et al. [48]	2007	22	73	50	-	100	23	0
Wicherts et al. [49]	2008	59	78	-	126	69	59	7
Homayounfar et al. [50]	2009	24	100	36	55	63	58	5
Tsai et al. [51]	2010	45	78	-	135	78	26	6
Brouquet et al. [52]	2011	65	70	-	56	72	49	6
Tsim et al. [53]	2011	38	95	-	-	87	33	0
Narita et al. [54]	2011	80	95	-	92	76	54	0
Muratore et al. [55]	2012	47	81	-	114	77	44	0
Turrini et al. [56]	2012	42	100	-	72	71	20	6
Shindoh et al. [57]	2013	144	98	62	34	72	58	6
Fuks et al. [58]	2015	34	15	-	93	76	50	3
Passot et al. [59]	2017	109	73	-	-	82	27	6

*PVE*, portal vein embolization; *PVL*, portal vein ligation

*Future liver remnant

°Time interval between interventions and the surgical resection

A major limitation of conventional TSH, but also one-stage resections with PVE, is the unpredictable long interstage interval [10]. Tumor progression after the first stage was the reason in 80% and insufficient liver growth in the remaining 20% for not proceeding to second-stage completion hepatectomy [54, 55, 63–65]. Three or more metastases in the FLR and an age over 70 years were predictive of tumor progression or development of *de novo* metastases in multivariate analysis [54]. These facts clearly show the importance to protect against tumor progression during the interstage interval. In order to counteract potential tumor growth, chemotherapy is commonly administered during the interstage interval. Interstage chemotherapy reduced the rate of disease progression without negatively affecting liver hypertrophy [64, 66]. Pathologic response to chemotherapy was associated with second-stage completion and longer survival [67]. In a series of CRLM from Memorial Sloan-Kettering Cancer Center, disease progression was observed in 34% after PVE without interstage chemotherapy as compared to 19% when chemotherapy was administered. OS was improved in patients receiving interstage chemotherapy with 50 versus 24 months [68].

## Associating liver partition with portal vein ligation for staged hepatectomy

**Fig. 6:**
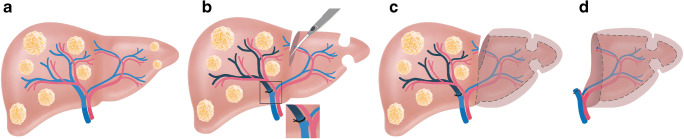
As an alternative to a conventional TSH, associating liver partition and portal vein ligation for staged hepatectomy (ALPPS) can be performed for extensive bilobar tumorload (**A**). In the first stage portal vein ligation, clearing of the FLR and liver transection is performed (**B**). After an interstage interval of 1–2 weeks (**C**), sufficient hypertrophy is observed and completion hepatectomy is performed (**D**)

**Fig. 7:**
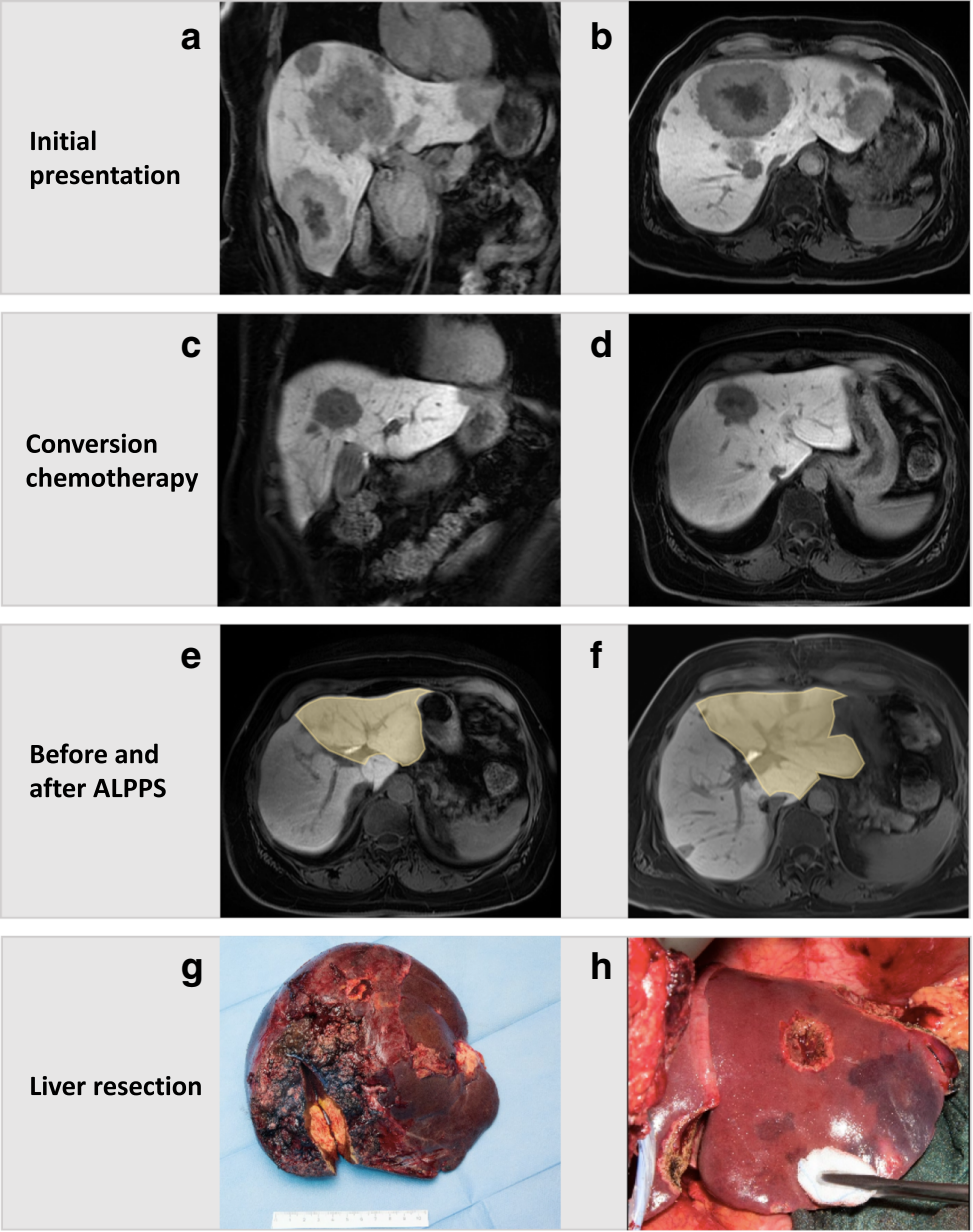
Clinical case of associating liver partition and portal vein ligation for staged hepatectomy (University of Zurich). We present the case of a 61-year-old patient with synchronous bilobar colorectal liver metastases (**A**/**B**). After neo-adjuvant therapy with folinic acid, oxaliplatin, fluorouracil (FOLFOX), and panitumumab, a liver first approach was chosen (**C**/**D**). Due to a small sFLR of 18% (**E**), we decided to perform a two-staged hepatectomy. In the first stage, clearing of the left hemi-liver with four atypical resections, 50% parenchymal transection, and right-sided portal vein ligation were performed (C). Eight days later, and after an increase of the sFLR to 29% (**F**), we performed completion hepatectomy (**G**) (**H**). Two months after ALPPS, the patient was scheduled for adjuvant chemotherapy

Combining PVL and parenchymal transection at the first stage results in an additional growth stimulus of the FLR making completion hepatectomy feasible in already 1–2 weeks (Fig. 6) [69–73]. This procedure was subsequently termed ALPPS [74]. In the inaugural German report, 25 patients underwent ALPPS for various primary and secondary liver tumors. After a median interstage interval of 9 days, a remarkable FLR hypertrophy of 74% was observed [69]. This acceleration of liver parenchymal augmentation has created some enthusiasm worldwide as completion rates (i.e., successful progression to stage 2 surgery) exceeded 95% in most series [75]. In other words, ALPPS was able to markedly increase the number of potentially curative resections compared to PVE and TSH (Fig. 7) [76]. This advantage of ALPPS has led to a universal application for patients with different tumor entities and risk profiles in many centers. The price for that was an initially high perioperative morbidity and mortality [77–84]. Comprehensive analyses based on the international ALPPS registry demonstrated that age ≥ 67 years, biliary tumors, major interstage complications (Clavien-Dindo ≥3b), and elevated bilirubin and creatinine before stage 2 were associated with 90-day or in-hospital mortality [85, 86]. Most centers subsequently refined their patient selection and were more cautious with proceeding with stage 2 surgery in case predictors for short-term mortality were present [80, 87]. In addition, technical modifications have led to a less invasivestage 1 surgery with the goal to enable an uncompromised liver parenchymal hypertrophy in the interstage interval in many centers. These technical modifications include partial-ALPPS [88, 89], laparoscopic ALPPS [90], tourniquet ALPPS [91], and mini-ALPPS [92]. An analysis form the international ALPPS registry found the combination of risk adjustment in patient selection and technique resulted in a significant decrease in early morbidity and mortality to levels which are widely accepted for major liver surgery [87]. With accumulating experience in some expert centers, ALPPS has been re-considered for non-CRLM tumor entities like neuroendocrine liver metastases [93]**,** intrahepatic CC [94], HCC [95, 96], and PHC [97] in selected cases. In an attempt to define reference values for ALPPS, a recent benchmark analysis focusing on a low-risk population with CRLM found a 90-day mortality rate ≤ 5%, major complications (Clavien-Dindo grade ≥ 3a) ≤65%, postoperative liver failure after stage 2: ≤5%, and completion of stage 2 surgery ≥96% [98]. Of note, in this population, the FLR increased from 0.21% (SD 0.12) to 0.41 (SD 0.09) after a median interstage interval of 13 days [98].

A Scandinavian multicenter RCT comparing ALPPS and TSH in 100 patients confirms the evidence from single, retrospective series and registry data that ALPPS is associated with significantly higher resection rates (ALPPS, 92% vs. TSH, 57%). In this trial, patients with CRLM and sFLR <30% were randomized into ALPPS versus TSH. The ALPPS group had a significant greater volume increase of 68% vs. 36%, while stage 2 was performed after 11 days in the ALPPS group compared to 43 days in the TSH group. Of note, 12 patients that failed to reach an sFLR of ≥30% in the TSH arm were successfully treated with rescue ALPPS. Interestingly, the study found no difference in major complications (ALPPS, 43% vs. TSH, 43%) and 90-day mortality (ALPPS, 8.3% vs. THS, 6.1%) [73]. The survival analysis of the RCT furthermore showed an improved median OS of 46 months for patients randomized to ALPPS versus 26 months randomized to TSH [99]. Looking at the hypertrophy induced by ALPPS in more detail, two meta-analyses found significantly greater hypertrophy for the ALPPS group compared to PVE. The study by Eshmuminov et al. included two studies and found an FLR increase of 76% in the ALPPS group versus 37 % in the PVE groups (*P* < 0·001) [75]. A more recent meta-analysis found that ALPPS induced a greater FLR increase compared to PVE (RR 6.30; 95%CI, 3.97–8.64) and conventional TSH (RR 3.27; 95%CI, 1.63–4.91) [100]. Studies comparing ALPPS versus conventional TSH with PVE and PVL are shown in Table 3.

**Table 3:** Comparative studies evaluating patient outcomes for PVE and ALPPS

Reference	Year	Study design	Strategy	Patients (*n*)	Volume increase* (%)	Time° (d)	Resection rate (%)
Knoefel et al. [70]	2012	RCS	ALPPS	7	63	8	100
			PVE	15	37	35	80
Tanaka et al. [83]	2015	RCS	ALPPS	10	54	-	-
			PVE	54	19	-	-
Croome et al. [71]	2015	RCS	ALPPS	15	84°°	8	100
			PVE	53	36°°	40	79
Schadde et al. [82]	2015	RCS	ALPPS	320	100°°	14	98
Ratti et al. [84]	2015	RCS	ALPPS	12	47	11	100
			PVE/PVL	36	41	31	94
Chia et al. [72]	2017	RCS	ALPPS	10	48	-	80
			PVE/PVL	29	12	46	59
Sandström et al. [73]	2018	RCT	ALPPS	48	68	11	92
			PVE/PVL	49	36	43	57
Chan et al. [95]	2019	RCS	ALPPS	46	48	7	98
			PVE	102	38	48	68

*ALPPS*, associating liver partition and portal vein ligation for staged hepatectomy; *PVE*, portal vein embolization; *RCS*, retrospective case series; RCT, Randomized controlled trial

*Future liver remnant

°Time interval between interventions and the surgical resection

°°Standardized future liver remnant.

## Resection and partial liver segment 2/3 transplantation with delayed total hepatectomy and associated concepts

**Fig. 8:**
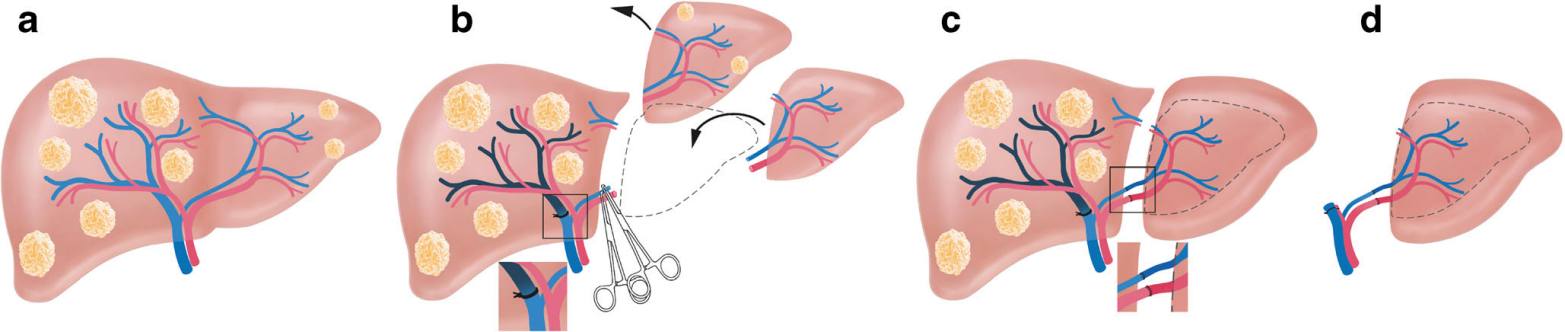
The RAPID approach combines resection with partial liver segment 2/3 transplantation and delayed total hepatectomy for bilobar liver metastases (**A**). In a first step, segments 1–3 are removed, in combination with right portal vein ligation (**B**) and transplantation of a left partial allograft (**C**). After sufficient regeneration, the native right hemi-liver is removed in a second step (**D**)

This innovative two-stage concept, introduced by the Oslo group in 2015, combines regeneration of a left lateral partial liver graft followed by delayed resection of the native, metastatic liver [101]. In a first stage, segments 1–3 are resected and a partial segment 2–3 graft from a deceased donor is transplanted orthotopically. In addition, the right portal vein is ligated in this stage directing the portal flow to the graft. After sufficient regeneration of the allograft, the native right hemi-liver is resected **(**Fig. 8). In other words, the deportalized native liver is turning its function into an auxiliary liver enabling the left lateral graft to regenerate in the interstage interval. Compared to whole organ liver transplantation, the RAPID procedure has the advantage that the precious donor pool is not affected as the extended right liver graft can be transplanted to adult patients without significant disadvantage [102]. To evaluate the clinical benefits of the RAPID procedure including step two completion rate and the overall survival, the Oslo group is currently recruiting patients for their prospective study (NCT02215889). To date, the experience is limited to 3 transplanted patients with one patient disease-free after 5.5 years, one alive after 2 years with lung recurrence after 12 months, and one mortality due to graft hepatic artery thrombosis 40 days post-RAPID [103].

Addressing the lack of split policy and low organ donation rates, Königsrainer et al. proposed living donor (LD) RAPID procedure [104]. To our knowledge, 8 LD-RAPID have been performed so far, 5 in Germany, 2 in Italy, and 1 in Belgium. Of note, three patients are alive and tumor-free after 6–18 months [103]. A prospective bi-institutional German trial (University of Tübingen and University of Jena) currently evaluates the feasibility, safety, and efficacy of LD-RAPID for non-resectable CRLM with overall survival at 36 months as primary endpoint (NCT03488953) [105].

**Fig. 9:**
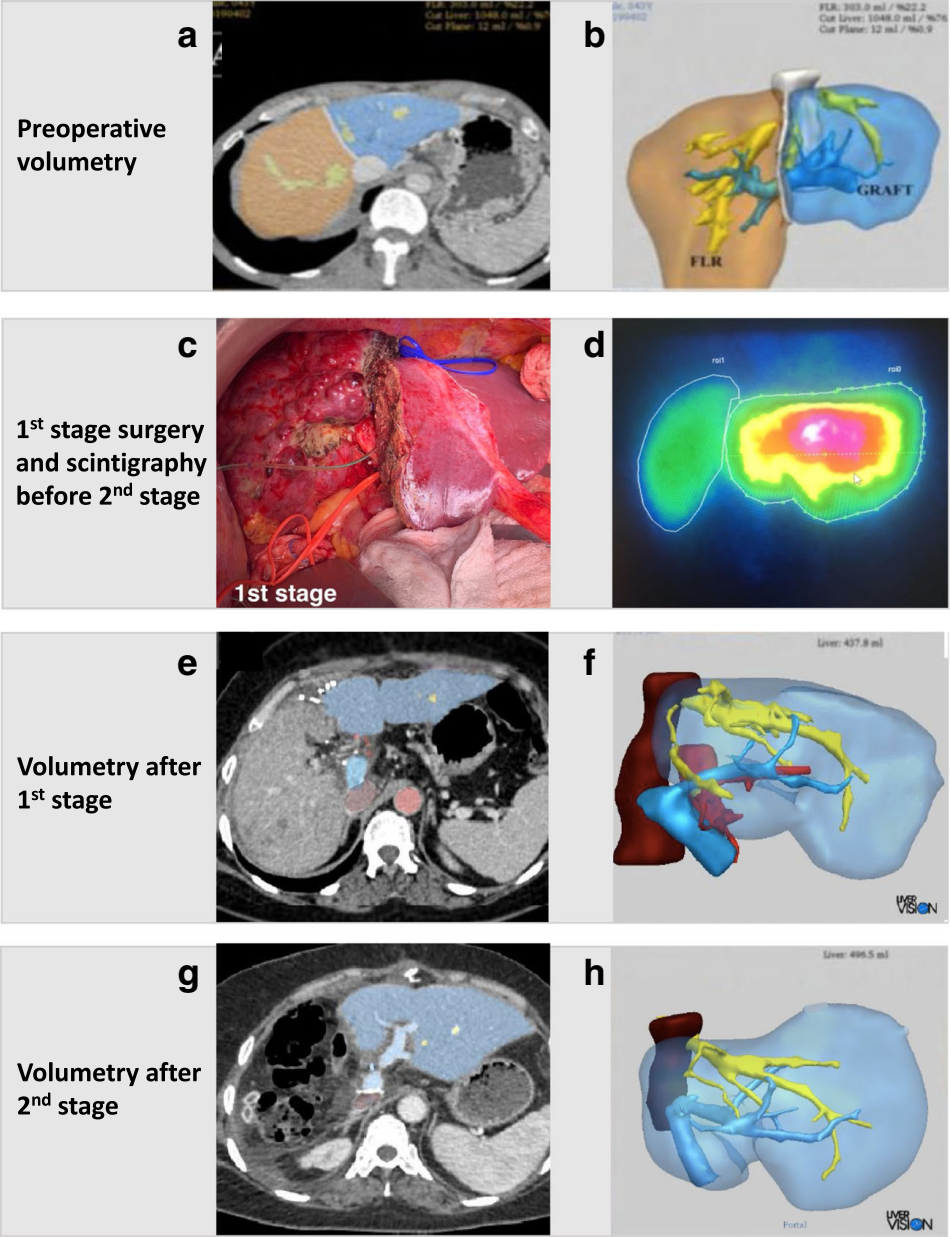
Clinical case of RAPID approach in a cirrhotic patient with portal hypertension and hepatocellular carcinoma (Ankara University School of Medicine) [106]. After removing segments 1–3 (**A**/**B**), a small left lobe graft (segments 2, 3, 4; graft-to-recipient weight ratio 0.35) was transplanted (**C**). Due to the portal hypertension, inflow modification was necessary and a hemiportocaval shunt was created that re-routed approximately two-thirds of portal flow to the inferior vena cava decreasing the portal pressure from 24 to 14 mmHg. After 22 days, hepatobiliary scintigraphy showed an almost 70% functional shift to the left lobe graft (**D**), and there was a 56% graft volume hypertrophy (**E**, **F**). In the second stage, the right hemi-liver was removed, with closure of the hemiportocaval shunt. To control potential portal hyperflow and arterial buffer response, splenic artery ligation was added at the end of the operation. The patient recovered without liver failure and is without tumor recurrence almost 2 years after the 1st-stage operation (**G**/**H**)

Recently, the RAPID procedure has also been described in a patient with liver cirrhosis, portal hypertension (PHT), and a 3 cm HCC, potentially extending the indication to selected patients with underlying liver disease [106]. Balci et al. used a small left lobe graft (graft-to-recipient weight ratio 0.35) for LD-liver transplantation. In order to overcome PHT in the cirrhotic setting, inflow modification was necessary to provide an adequate portal- and hepatic artery flow to the partial graft, while decreasing portal pressure to prevent early allograft dysfunction. A hemiportacaval shunt was created, which re-routed two-thirds of the portal flow to the inferior vena cava, decreasing portal pressure from 24 to 14 mmHg in the first stage. Twenty-two days after the first operation, there was a 56% graft volume increase and a functional shift of over 60% to the partial graft. At the end of second operation, the hemiportacaval shunt was closed and the splenic artery ligated in order to control for potential portal hyperflow and arterial buffer response. The patient recovered without liver failure and is without tumor recurrence almost 2 years after the operation (Fig. 9) [106].

Taking the concept of RAPID further, the Bologna group has recently reported the transplantation of such graft heterotopically into the splenic fossa [107, 108]. This procedure is coined “hete**R**otopic tr**A**nsplantation of segments 2 and 3 using the splenic **V**ein and **A**rtery after **S**plenectomy and with delayed total hepatectomy“ (RAVAS) and necessitates a splenectomy before partial liver transplantation can be performed. The authors describe one case of a 40-year-old male with synchronous, unresectable CRLM. The original plan was to perform a TSH with cleaning of the FLR and subsequent PVE. This plan, however, did not succeed as the patient developed a severe bile leak and could not undergo completion hepatectomy. The authors utilized a left lateral graft, which was rejected for pediatric liver transplantation. In contrast to RAPID, the authors placed a tourniquet on the main PV of the native liver to modulate portal flow to the graft. After an interstage interval of 2 weeks, the graft-to-body weight ratio successfully augmented from 0.6 to 1 and native hepatectomy became feasible. At 8 months after RAVAS, the patient is in good health condition with no evidence of tumor recurrence. A potential advantage of this procedure is to avoid manipulation of the native, tumor-bearing liver. In the past, however, heterotopic liver transplantation has turned out to be prone to vascular complications with kinking of vessels and particularly an unfavorable outflow situation with a high risk of Budd Chiari syndrome [109].

Common to RAPID and associated concepts is that they may increase the employment of liver transplantation in metastatic disease in selected cases. The major advantage is that small segment 2/3 grafts can be augmented to a fully functioning graft with the temporary help of an auxiliary, deportalized native liver. These procedures probably rank among the most complex procedures in liver surgery requiring profound experience of surgeons and the whole care team in extensive liver surgery and living donor liver transplantation.

## Conclusion

Since the introduction of the first liver parenchyma augmentation–assisted techniques of PVE in the 1980s, this field has tremendously evolved meanwhile creating an own established niche in liver surgery. Utilizing the unique ability of the liver to regenerate enables most extended resections with leaving only a very small liver remnant behind. Nevertheless, most of the techniques rely on portal vein occlusion, but more recently inclusion of parenchymal splitting, hepatic vein occlusion, and partial liver transplantation has extended the technical armamentarium to induce liver parenchymal augmentation prior hepatectomy. Safely accomplishing major and ultimately total hepatectomy by this means requires an integration into a meaningful oncological concept. The availability of highly effective chemotherapeutic regimens in the neo-adjuvant, interstage, and adjuvant setting underlines an aggressive surgical approach in the given tumor setting to convert formerly “palliative” disease into a curative and sometimes in a “chronic” disease.

## Abbreviations

ALPPS: Associating liver partition and portal vein ligation for staged hepatectomy
CRLM: Colorectal liver metastasis
DFS: Disease-free survival
FLR: Future liver remnant
HCC: Hepatocellular carcinoma
HV: Hepatic vein
HVE: Hepatic vein embolization
LD: Living donor
PHC: Perihilar cholangiocarcinoma
PHLF: Post-hepatectomy liver failure
PHT: Portal hypertension
PV: Portal vein
PVE: Portal vein embolization
PVL: Portal vein ligation
OS: Overall survival
RAPID: Resection and partial liver segment 2/3 transplantation with delayed total hepatectomy
RASPE: Radiological simultaneous portohepatic vein embolization
RCT: Randomized controlled trial
TACE: Transarterial chemoembolization
TSH: Two-staged hepatectomy
